# Interhemispheric asymmetry during NREM sleep in the dog

**DOI:** 10.1038/s41598-021-98178-3

**Published:** 2021-09-22

**Authors:** Vivien Reicher, Anna Kis, Péter Simor, Róbert Bódizs, Márta Gácsi

**Affiliations:** 1 Department of Ethology, Institute of Biology, Eötvös Loránd University, Budapest, Hungary; 2 MTA-ELTE Comparative Ethology Research Group, Budapest, Hungary; 3 Institute of Cognitive Neuroscience and Psychology, Research Centre for Natural Sciences, Budapest, Hungary; 4 Institute of Psychology, Eötvös Loránd University, Budapest, Hungary; 5 Institute of Behavioural Sciences, Semmelweis University, Budapest, Hungary; 6 National Institute of Clinical Neurosciences, Budapest, Hungary

**Keywords:** Neuroscience, Circadian rhythms and sleep

## Abstract

Functional hemispheric asymmetry was evidenced in many species during sleep. Dogs seem to show hemispheric asymmetry during wakefulness; however, their asymmetric neural activity during sleep was not yet explored. The present study investigated interhemispheric asymmetry in family dogs using non-invasive polysomnography. EEG recordings during 3-h-long afternoon naps were carried out (N = 19) on two occasions at the same location. Hemispheric asymmetry was assessed during NREM sleep, using bilateral EEG channels. To include periods with high homeostatic sleep pressure and to reduce the variance of the time spent in NREM sleep between dogs, the first two sleep cycles were analysed. Left hemispheric predominance of slow frequency range was detected in the first sleep cycle of sleep recording 1, compared to the baseline level of zero asymmetry as well as to the first sleep cycle of sleep recording 2. Regarding the strength of hemispheric asymmetry, we found greater absolute hemispheric asymmetry in the second sleep cycle of sleep recording 1 and 2 in the frequency ranges of alpha, sigma and beta, compared to the first sleep cycle. Differences between sleep recordings and consecutive sleep cycles might be indicative of adaptation-like processes, but do not closely resemble the results described in humans.

## Introduction

Functional asymmetry of the brain occurs if the right and left hemispheres intercede different behavioural and cognitive processes^1^. This phenomenon generally exists in a wide range of non-human species, including insects, fish, amphibians and birds^2–6^.

One crucial sub-type of hemispheric asymmetries occurs during sleep. The strongest hemispheric asymmetry is known to appear in sleeping aquatic mammals and birds (for review^7,8^). To keep a constant vigilance state, these animals manifest a sleep behaviour, known as unihemispheric sleep, during which one cerebral hemisphere expresses sleep-like neural activity while the other shows wake-like neural activity^7,8^. Terrestrial mammals, including humans, do not show obvious signs of unihemispheric sleep. Electroencephalic (EEG) sleep studies, however, proved that small hemispheric asymmetries can still be detected^7^, but their function is far from being clarified. According to an ecological theory, hemispheric asymmetry in humans might help to monitor the external environment during sleep^9^, resembling the function of unihemispheric sleep in aquatic mammals and birds that need maintain (a much higher level of) alertness during sleep states. In humans, results suggest that the first-night effect, which manifests in marked differences in sleep macrostructure between the first and second sleep occasions^10,11^, is probably linked to asymmetric sleep: one hemisphere is more vigilant than the other during sleep in order to monitor the unfamiliar environment^9^. Several environmental factors play a role in eliciting the first-night effect^11^. It is assumed that evolutionary adaptations have shaped humans’ sleep (e.g. sleeping in a protected environment might have led to increased deepness of human sleep)^12^. Due to these considerations, it would be relevant to examine if the sleep of other species adapted to the human environment (e.g. domestic animals such as the dog) is also characterised by hemispheric asymmetries.

There is a recent interest in studying behavioural and brain asymmetry in the dog^13–17^. In dogs, the sensory and motor asymmetry have been extensively investigated (See review^13,14^). Recently, neural studies using fMRI revealed asymmetrical activation in the dog brain. For example, right hemispheric lateralization was detected when processing human words^18^, thermal radiation elicited increased neural response in the left somatosensory cortex^19^.

In addition to the investigation of awake functioning with fMRI (e.g.^20,21^) and EEG (e.g.^22,23^), dogs’ natural sleep architecture has been widely studied by non-invasive polysomnography (PSG) adapted to dogs (for review^24,25^). This method has been successfully conducted in several studies investigating the sleep architecture with pre-sleep experiences, such as memory consolidation^26^, emotion processing^27^ and sensitivity to the location and timing of sleep^28^. Nevertheless, few studies examined the natural sleep of dogs without including experimental manipulations before sleep^29,30^, but neither of these provided information about asymmetrical brain activity.

In this study we examined the dogs’ hemispheric asymmetry during daytime sleep. We focused on the first two cycles of NREM sleep due to several reasons. Dogs exhibit a great variance regarding the time spent in different sleep stages, not only between but within sleep occasions^30^. Furthermore, they spend more time in NREM sleep in the first hour of sleep recordings, compared to the next two hours^28^. To reduce the potential variance of the time spent in NREM sleep between dogs, we analysed only the first two sleep cycles of each dog. The reason for focusing solely on NREM sleep is that the EEG recorded in this stage provides the most artefact-free traces, which is crucial for the current study using data from two sleep cycles only. Apart from this practical and methodological consideration, a further theoretical argument for focusing on NREM sleep is that EEG slow wave activity in NREM is a reliable marker of sleep intensity not just in humans^31^ but in other animal species as well (e.g., rat^32^; hamster^33^; mouse^34^).

In sum, our aim was to provide a descriptive analysis on the asymmetrical hemispheric activation in the dog’s natural sleep. To test this, we explored whether the direction and strength of interhemispheric asymmetry changes between consecutive sleep cycles and sleep recordings.

## Method

### Subjects

Our subjects were N = 30 family dogs who attended our Canine EEG lab with their owners. Sleep data of these dogs were included in our previous sleep study that focused on macrostructural sleep parameters (Reicher et al. 2020). Dogs that did not have at least six minutes of artefact-free NREM sleep during the recordings, e.g. due to lack of NREM sleep and strong muscle tone (N = 11) were excluded. The 19 dogs whose data were used for the analysis were 1–9 years old (mean age = 4.6 + /− 2.4); from 8 different breeds and 8 mongrels; 8 males (3 neutered) and 11 females (9 neutered), and no female dogs were in heat during any of the recording occasions).

The experiment was carried out in accordance with the Hungarian regulations on animal experimentation and the ‘Guidelines for the use of animals in research’ described by the Association for the Study of Animal Behavior (ASAB). Our experimental protocol was approved by the Scientific Ethics Committee for Animal Experimentation of Budapest, Hungary, by categorizing it as a non-invasive study (No. of approval: PE/EA/853–2/2016).

### Procedure

Owners were recruited from the Family Dog Project (Eötvös Loránd University, Department of Ethology) database, and prior to the onset of the experiment, they provided their informed consent. The location of the measurements was a fully equipped laboratory for EEG measurements at the Research Centre for Natural Sciences, Institute of Cognitive Neuroscience and Psychology (see Reicher 2020 for the details of the laboratory arrangement).

Participation in the sleep EEG research did not require prior training. All subjects were measured on two occasions at the same location. All the recordings were conducted during the afternoon, with a start time between 12 and 5 pm. For each individual dog, the starting time of the two different nap opportunities was kept within a ± 2-h interval between recording occasions. The time interval between two consecutive sleep recordings was: mean week = 3.9 ± 1.6.

### Polysomnographic method

**Figure 1:**
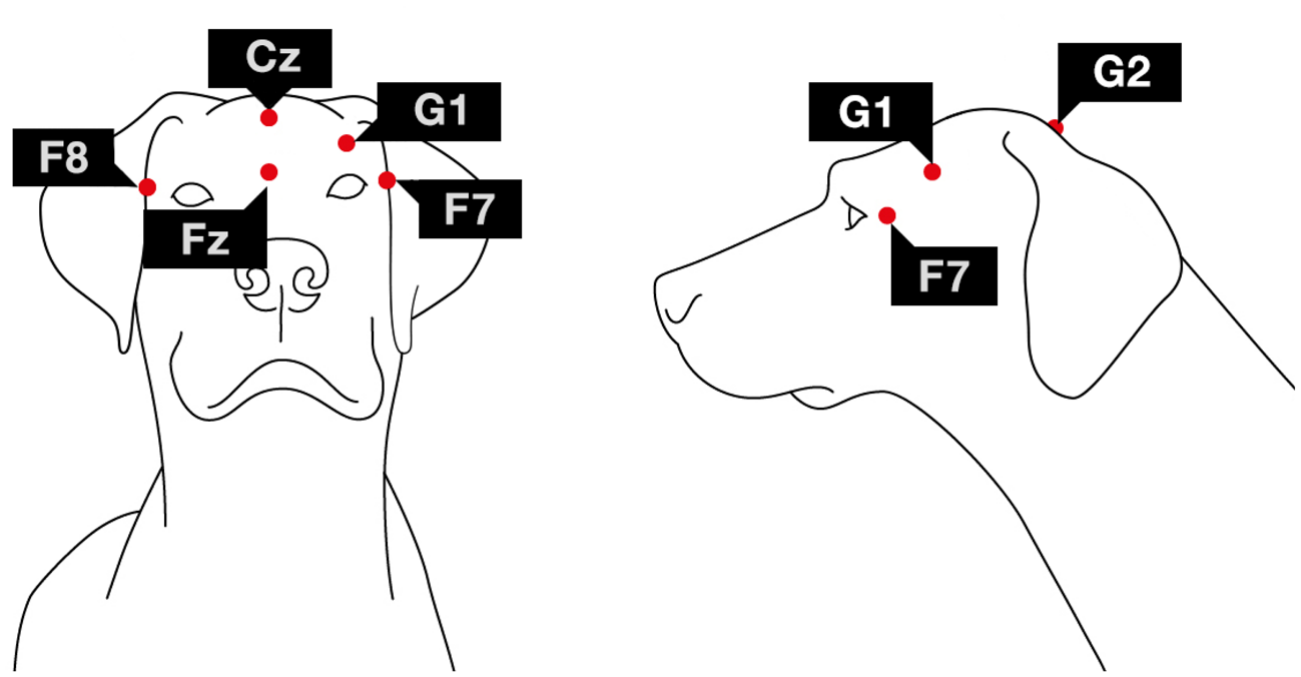
Placement of the electrodes (Fz-Cz: frontal and central midline; F8 and F7: right and left electrodes placed on the zygomatic arch; G2: reference electrode; G1: ground electrode). EEG traces of different sleep stages and a wake period is included in our Supplementary document (Fig. S1).

A detailed description of the most recent polysomnographic method and EEG electrode placement (Fig. 1) can be found in Reicher et al. (2020). The current study used four active EEG channels (F7, F8, Fz, Cz), all referred to G2. Two electrodes were placed on the right and left zygomatic arch next to the eyes (F8, F7) and the scalp electrodes over the anteroposterior midline of the skull (Fz, Cz). The reference electrode (G2) was placed in the posterior midline of the skull (occiput; external occipital protuberance). The ground electrode (G1) was attached to the left musculus temporalis. In addition, ECG, EMG and respiration were also recorded for the purpose of hypnogram scoring (see later) and an additional channel, labelled EOG, was visualised as the bipolarly referenced F7–F8 electrodes. ECG electrodes were placed bilaterally over the second rib and EMG electrodes were placed bilaterally on the musculus iliocostalis dorsi. Respiration was recorded via a chest respiratory belt. The signals were collected, pre-filtered, amplified and digitized at a sampling rate of 1024 Hz/channel, using the 25 channel SAM 25R EEG System (Micromed, Mogliano Veneto, Italy), and the System Plus Evolution software with second-order filters at 0.016 Hz (high pass) and 70 Hz (low pass). EEG traces of different sleep stages and a wake period is included in our Supplementary documen﻿t (Fig. S1).

### Data analysis

Sleep recordings were visually scored in 20 s epochs in accordance with standard criteria^35^, adapted for dogs^29^ using Fercio’s EEG Plus (© Ferenc Gombos 2009–2021). This method is suitable for the reliable identification of the stages of wake, drowsiness, NREM and REM^36^, thus resulting in a hypnogram for each recording. Following the hypnogram scoring, the first two sleep cycles were identified for the purpose of further analysis.

#### Definition of sleep cycle in the dog

**Figure 2:**
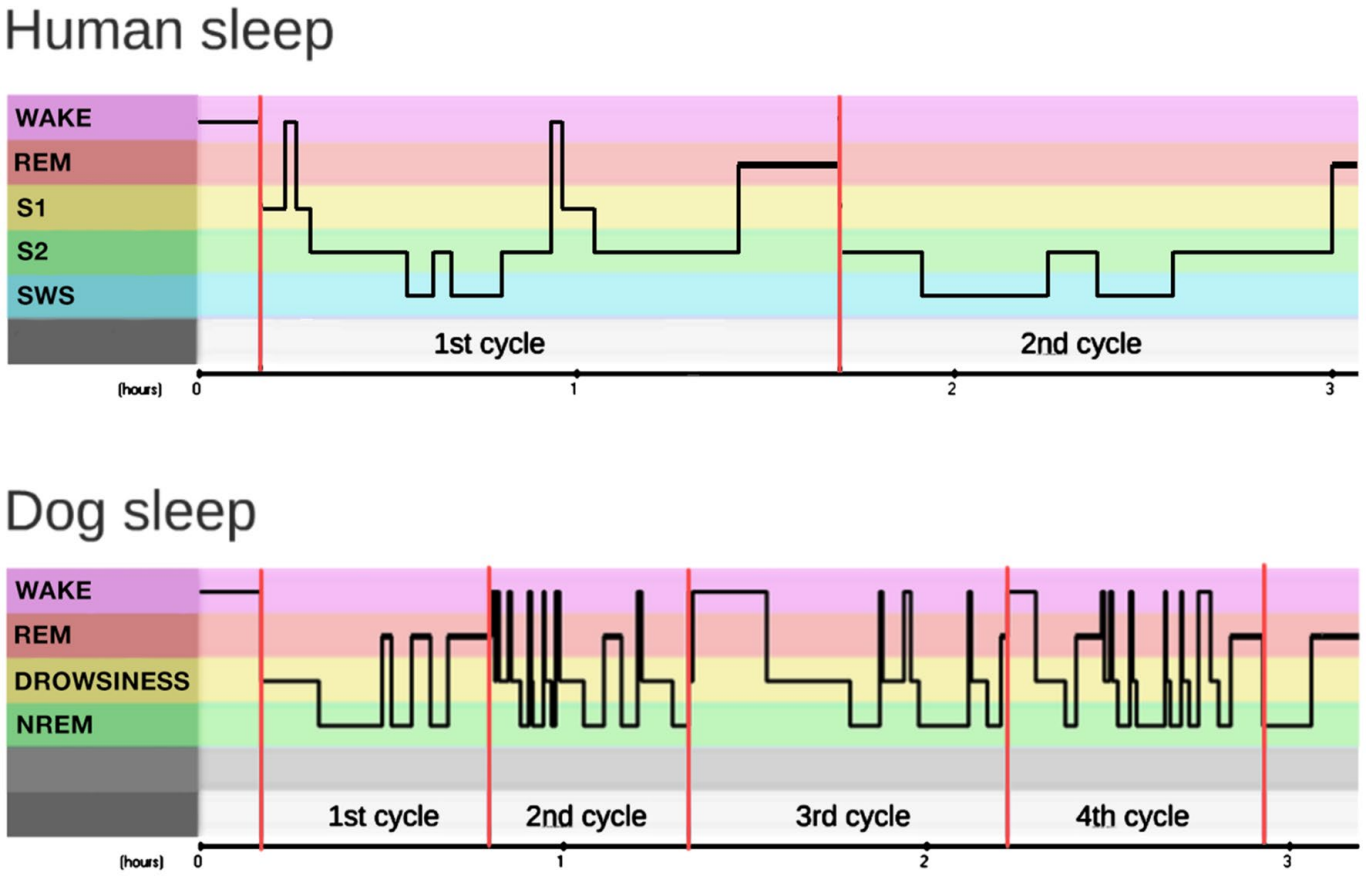
Typical hypnogram of three-hour-long sleep recordings of a human and a dog. In dogs due to the great number of awakenings and short REM sleeps compared to humans, we defined a dog’s sleep cycle differently. Our criteria were that (1) a sleep cycle should contain at least 6 min of NREM sleep, (2) if a wake period contained more than 5 min wake time, the subsequent sleep period was defined as the beginning of the next sleep cycle, (3) if a sleep cycle ended with a REM phase longer than 3 min and an awakening (of any length) followed, the subsequent sleep period was also defined as the next sleep cycle. We provided descriptive data of sleep macrostructure (mean ± SD durations of drowsiness, NREM and REM sleep) of each sleep cycle in sleep recording 1 and 2 (see our Supplementary document, Table S1).

In dogs, the definition of a sleep cycle is somewhat different from the rules applied in humans^37^. In humans, sleep cycles are a clearly delineated succession of different stages of NREM sleep and a REM sleep episode that closes the cycle. This is followed by a subsequent NREM phase, which is already part of the next sleep cycle^38^. As dogs display polyphasic sleep^39^, their sleep pattern is more variable and fragmented by frequent awakenings, compared to that of humans (see Fig. 2 as an example). As a consequence, dogs’ REM sleep is typically short and/or left out and NREM phases are intermittent with shorter/longer awakenings. While in humans, REM is most often followed by NREM sleep, dogs and other other mammals are more likely to wake up after REM sleep, potentially reflecting an evolutionary adaptation to limit the time spent in a vulnerable state^12,40^. To define a sleep cycle in case of dogs, our criteria were that (1) a sleep cycle should contain at least 6 min of NREM sleep, (2) if a wake period contained more than 5 min of wake time, the subsequent sleep period was defined as the beginning of the next sleep cycle, (3) if a sleep cycle ended with a REM phase longer than 3 min and an awakening (of any length) followed, the subsequent sleep period was also defined as the next sleep cycle.

#### Sleep analysis

Artefact rejection was carried out manually on 4 s epochs before further automatic analyses on the first two sleep cycles of all recordings. To include a sleep cycle in our analysis, NREM sleep had to contain at least 6 min of artefact-free traces (parts of the recordings that did not contain heart rate artefact on the EEG channels or not clear EEG signals, etc.) to provide reliably enough data. Average power spectral densities (1–30 Hz) were calculated by a Fast Fourier Transformation (FFT) algorithm, applied to the 50% overlapping, Hanning-tapered 4 s windows of the EEG signal of the F7-G2 and F8-G2 derivations. Dogs are known to show notable individual-level variation in morphological features with regard to head musculature and skull shape and thickness^41^ that might have influenced the EEG data. To circumvent measurement error that might arise from these differences, absolute power was normalized by computing the relative power spectra. The asymmetry indices (AI) were calculated based on the EEG spectral power of F7 and F8 channels during the NREM sleep of the first and the second sleep cycle. We calculated AI for each dog in each frequency bin by dividing the difference between the spectral power values of F7 and F8 channels by their sum: LI = (F7 − F8)/(F7 + F8).

#### Statistical analysis

To reveal the direction of hemispheric asymmetry, we performed analyses on AI values. Next, to explore the strength of hemispheric asymmetry, we performed analyses on the absolute asymmetry index values (ABS-AI).

Data analyses were performed in MATLAB (version 9.3.0.713579, R2017b, The MathWorks, Inc., Natick, MA) using the Fieldtrip open source toolbox^42^. Bin-wise spectrograms of ABS-AI and AI values across conditions (different sleep occasions and sleep cycles) were contrasted by permutation tests suitable to analyse EEG time series^43,44^. First, in order to explore variations in hemispheric asymmetry, we used two-sided paired t-tests to examine the differences between AI values and the baseline level of zero asymmetry in each frequency bin. Second, with two-sided paired t-tests we examined the differences between the AI values of sleep cycles and sleep recordings, as well as between the ABS-AI values of sleep cycles and sleep recordings. (ABS-AI values were not compared to the baseline level of zero asymmetry, since the absolute scores by definition deviate from zero).

In order to evaluate statistical significance, the observed t-values were compared against the distribution of t-values obtained from 1000 samples where the dependent measures (AI and ABS-AI values) were randomly shuffled across conditions. Statistical significance was set to *p* < 0.05. Non-parametric permutation statistics is an efficient way to analyse spectral power without making prior assumptions regarding the distribution of the data. Since we contrasted AI and ABS-AI values in each frequency bin, we had to consider the issue of multiple comparisons. In order to control for multiple comparisons, the so-called Rüger's areas^45^ were delineated the same way as in previous sleep studies^26,29^. As a consequence, sets of frequency bins with conventionally significant (*p* < 0.05) results regarding LI were accepted or rejected as significant as a whole. After defining these areas of neighbouring, consecutive frequency bins which contain a significant result, the number of significant results within the area was calculated. If at least half of these p values were below 0.025 (half of the conventional *p* = 0.05) and at least one third of them were below 0.0167 (one third of the conventional *p* = 0.05), the area as a whole was considered significant, otherwise, the area was considered non-significant.

## Results

We compared the durations of sleep stages (drowsiness, NREM and REM) in the first and second sleep cycles and in sleep recording 1 and 2. We found no differences between sleep cycles and sleep recordings (for results, see Supplementary Table S2).

### Direction of hemispheric asymmetry

**Figure 3:**
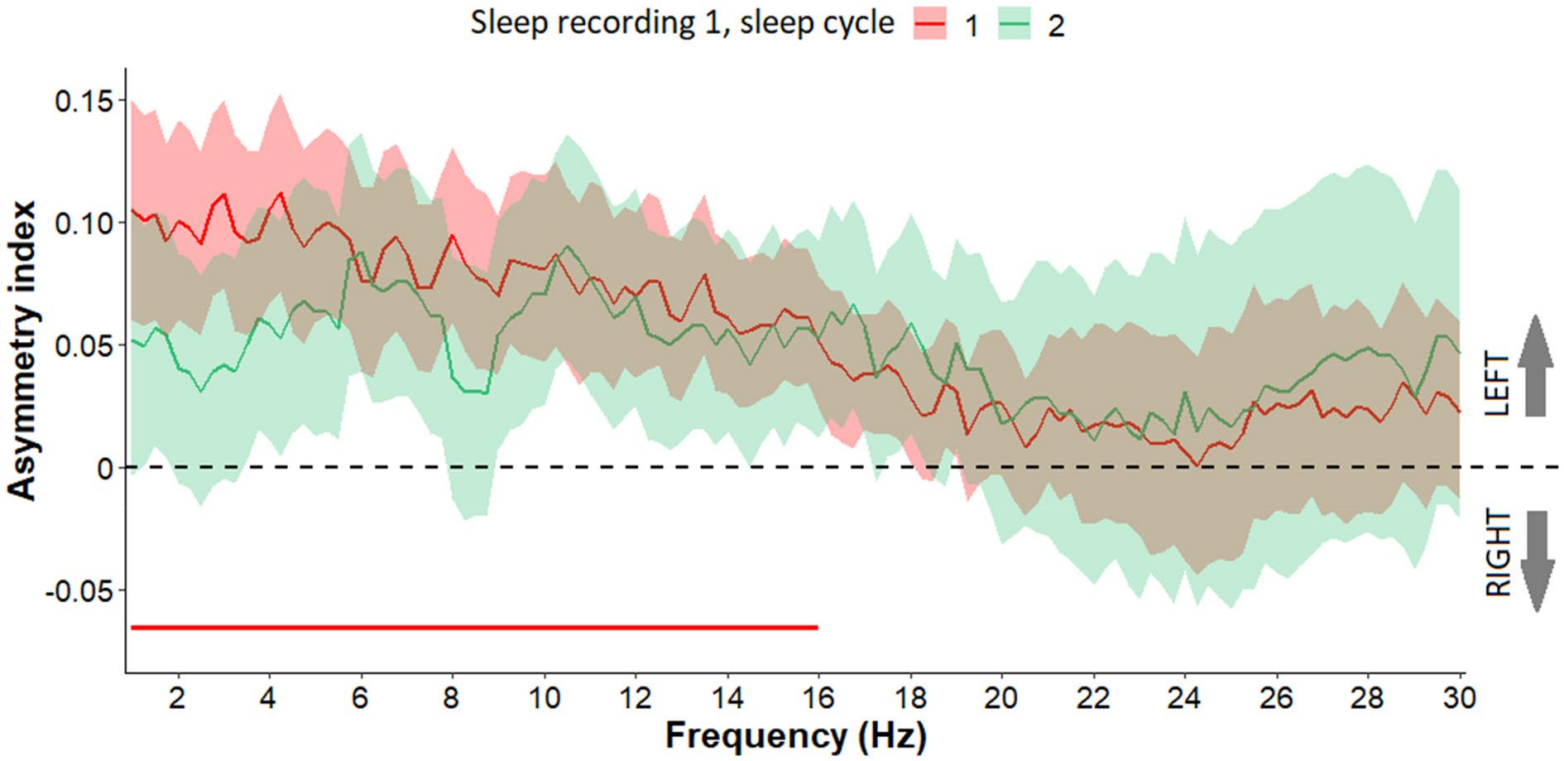
Asymmetry index values (Mean + / − SE) in frequency range 1–30 Hz in the first and second sleep cycle of sleep recording 1. Compared to baseline asymmetry (hypothetical zero), left hemispheric predominance was observed in the first sleep cycle of sleep recording 1 in the frequency range 1–12.75 Hz. Frequency bins below the statistical threshold (all *p*s < 0.05, fulfilling the criteria of a significant Rüger area) are illustrated by a red horizontal line.

We compared the first and second sleep cycles of sleep recording 1 to the baseline level of zero asymmetry. Left hemispheric predominance was observed in the first sleep cycle in the frequency range of 1–16 Hz (all *p*s < 0.05, fulfilling the criteria of a significant Rüger area in these frequency bins) (Fig. 3) but not in the second sleep cycle (all *p*s > 0.05). We considered the frequency range of 1–16 Hz significant, as the whole sets of consecutive frequency bins were conventionally significant (*p* < 0.05). Furthermore, half of these p values were below 0.025 and at least one third of them were below 0.0167. Thus, this area fulfilled the criteria of a significant Rüger area in these frequency bins. Regarding the comparison of the first and second sleep cycles to each other, we observed no significant differences (all *p*s > 0.05).

**Figure 4:**
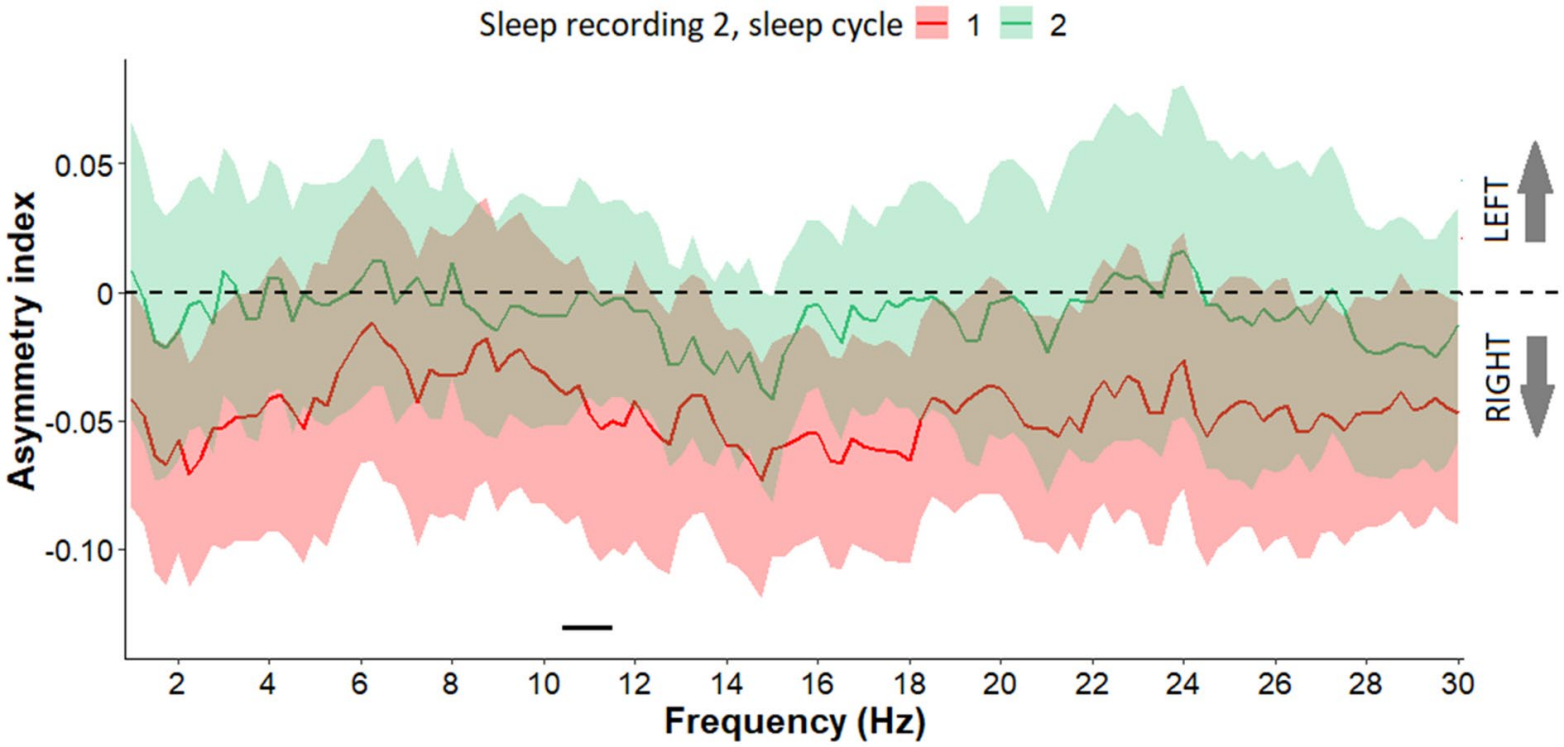
Asymmetry index values (Mean + / − SE) in frequency range 1–30 Hz in the first and second sleep cycle of sleep recording 2. In the first sleep cycle in frequency range of 10.5–11.5 Hz greater rightward asymmetry was observed, compared to the second sleep cycle of sleep recording 2. Frequency bins below the statistical threshold (all *p*s < 0.05, fulfilling the criteria of a significant Rüger area) are illustrated by a black horizontal line.

In sleep recording 2, the first and second sleep cycles did not deviate from the baseline asymmetry (all *p*s > 0.05) but the first sleep cycle showed greater rightward asymmetry in frequency range of 10.5–11.5 Hz, compared to the second sleep cycle (Fig. 4).

**Figure 5:**
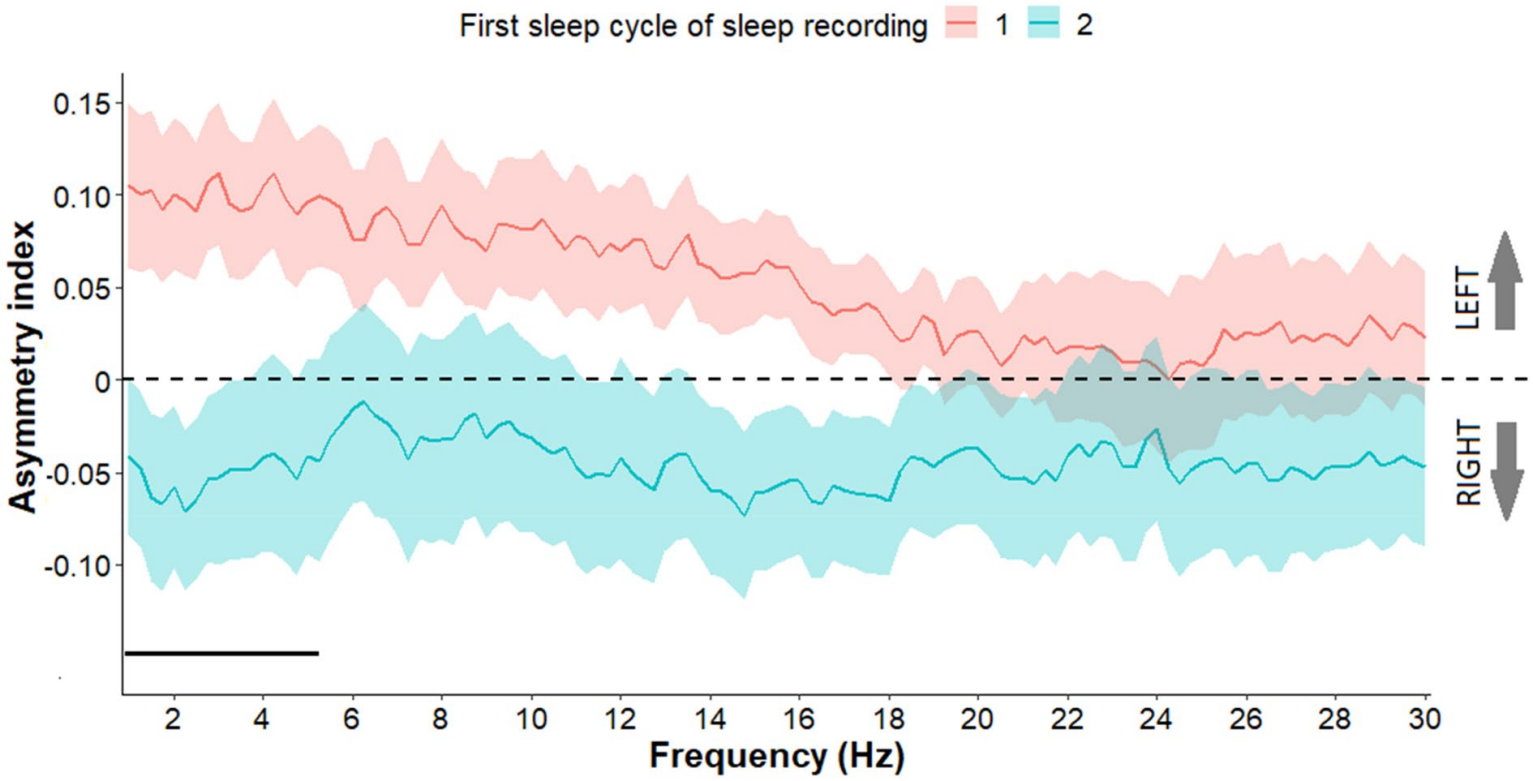
Asymmetry index values (Mean + / − SE) in frequency range 1–30 Hz in the first sleep cycles of sleep recording 1 and 2. In sleep recording 1 greater rightward asymmetry was observed in the frequency range of 1–5.25 Hz, compared to the sleep recording 2. Frequency bins below the statistical threshold (all *p*s < 0.05, fulfilling the criteria of a significant Rüger area) are illustrated by a black horizontal line.

We further compared the first sleep cycles of sleep recording 1 and 2 as well as the second sleep cycles of sleep recording 1 and 2. Regarding the comparison of the first cycles, we found greater leftward asymmetry in sleep recording 1 in the frequency range 1–5.25 Hz, compared to sleep recording 2 (all *p*s < 0.05) (Fig. 5). We observed no difference between the second cycles of sleep recording 1 and 2 (all *p*s > 0.05) (see Supplement for Fig. S2).

Since the first sleep cycle of sleep recording 1 significantly deviated from the hypothetical zero asymmetry as well as from the first cycle of sleep recording 2, we further examined whether the left hemispheric predominance in the low frequency band showed a relation with the degree of the first-night effect (FNE). Specifically, we examined whether the sum of asymmetry indices in the frequency bands of delta, theta, alpha and sigma are associated with the reduced sleep efficiency (sensitive marker of the first night effect in dogs^30^) in sleep recording 1. Kendall rank correlation revealed no significant associations (all *p*s > 0.05).

### Strength of hemispheric asymmetry

**Figure 6:**
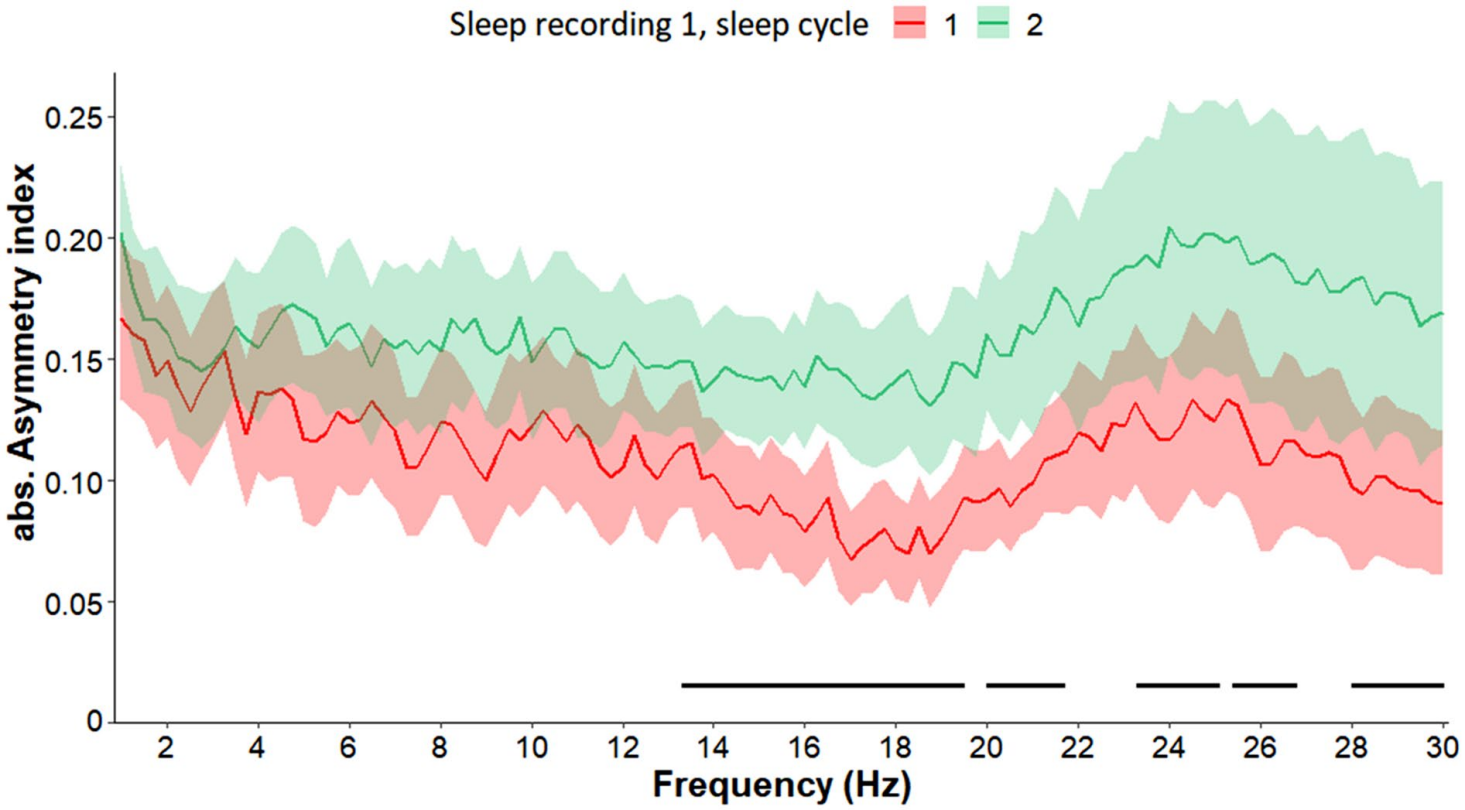
Absolute asymmetry index values (Mean + / − SE) in frequency range 1–30 Hz in the first and second sleep cycle of sleep recording 1. In the second sleep cycle greater asymmetry was observed in the frequency ranges of 13.5–19.5, 20–21.75, 23.5–25, 25.5–26.75 and 28–30 Hz, compared to the first sleep cycle. Frequency bins below the statistical threshold (all *p*s < 0.05, fulfilling the criteria of a significant Rüger area) are illustrated by a black horizontal line.

First, we compared the first and second sleep cycles of sleep recording 1 and found greater absolute asymmetry index values in the second sleep cycle in the frequency ranges of 13.5–19.5, 20–21.75, 23.5–25, 25.5–26.75 and 28–30 Hz (all *p*s < 0.05 fulfilling the criteria of a significant Rüger area in these frequency bins) (Fig. 6). Second, we compared the first and second sleep cycles of sleep recording 2 and observed no difference between the cycles (see Supplement for Fig. S3).

**Figure 7:**
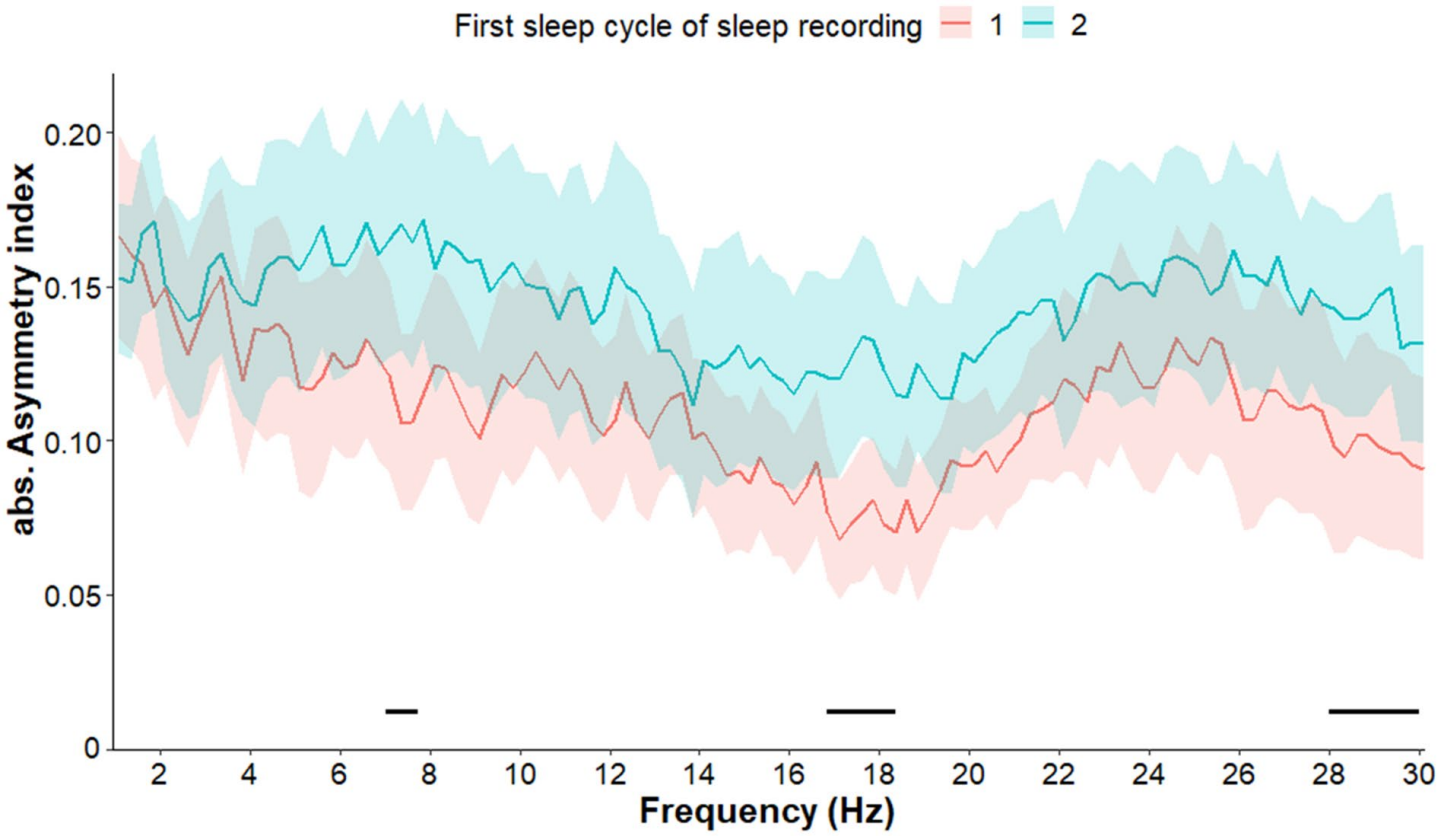
Absolute asymmetry index values (Mean + / − SE) in frequency range 1–30 Hz in the first sleep cycles of sleep recording 1 and 2. In the second sleep cycle greater asymmetry was observed in the frequency ranges of 7–7.75, 16.75–18.25 and 28–30 Hz, compared to the first sleep cycle. Frequency bins below the statistical threshold (all *p*s < 0.05, fulfilling the criteria of a significant Rüger area) are illustrated by a black horizontal line.

We further compared the first sleep cycles of sleep recording 1 and 2 as well as the second sleep cycles of sleep recording 1 and 2. Regarding the comparison of the first cycles, we found greater asymmetry in the second sleep cycle in the frequency ranges of 7–7.75, 16.75–18.25 and 28–30 Hz (all *p*s < 0.05 fulfilling the criteria of a significant Rüger area) (Fig. 7). We observed no difference between the second cycles of sleep recording 1 and 2 (all *p*s > 0.05) (see Supplement for Fig. S4.

### Individual variability

Furthermore, it is important to note that dogs showed huge individual variability. For example, while at the population level brain activity showed leftward asymmetry in sleep recording 1, at the individual level, 5 out of the 30 dogs deviated from this pattern (See Supplement, Fig. S5).

## Discussion

With this descriptive study we aimed to contribute to the universally described, but poorly understood phenomenon of hemispheric asymmetry. Using a fully non-invasive polysomnography method, we observed interhemispheric differences in family dogs’ sleep EEG spectrum during repeated afternoon recordings.

We found interhemispheric differences regarding the direction of hemispheric asymmetry. Leftward hemispheric predominance was detected in the frequency range of 1–12.75 Hz in the first sleep cycle of sleep recording 1, which deviated from the baseline level of zero asymmetry as well as from the first cycle of sleep recording 2. Similar effect was observed in sleep recording 2, but the first sleep cycle showed greater rightward asymmetry in a narrow frequency range (10.5–11.5 Hz), compared to the second sleep cycle. In humans, a complex pattern of interhemispheric asymmetry was reported, which was dependent on frequency ranges, EEG derivations, sleep pressure, age and sex^46–50^. For example, findings indicated greater delta activity (measured with delta waves counts) in the right frontal and central regions as compared to the corresponding left hemispheric regions^47^, whereas prolonged wakefulness was followed by an increase in left hemispheric enhancement of delta power^49^. Sigma activity was also greater in the right frontal regions, but a reverse pattern was reported for the parietal and occipital regions^48^. Likewise, studies using fMRI and MEG neuroimaging techniques found region-specific hemispheric asymmetries as well^9,51^.

The strength of EEG asymmetry during NREM sleep is largely variable across species. In our study, dogs had an average absolute asymmetry index value of 0.15, also showing notable individual variance ranging from 0 to 0.6. Based on previous reports, aquatic mammals showed the greatest absolute hemispheric asymmetry (e.g., in pinnipeds it reached 0.8^52^), while birds and rats exhibited smaller absolute asymmetry indices (in birds < 0.3^53^; in rats < 0.1^54,55^. In humans, the asymmetry was present with even smaller magnitude, with maximum absolute asymmetry index value of 0.1 measured with MEG^9^. The majority of non-human studies used invasive EEG techniques assessing data from implanted electrodes, causing potential further differences on the magnitude of hemispheric asymmetry. Of further note, Lyamin et al. highlights that in case of rats and birds data were scored in 4-s epochs, while other studies used 20-s epochs for calculation. This might also cause potential differences in EEG power between the two hemispheres^52^.

In the current study we analysed relative EEG power spectra to minimize the effect of notable individual-level variation in morphological features of dogs, however, future studies with larger and/or more homogeneous samples will need to assess whether dogs’ EEG spectrum values are dependent on muscle and/or skull thickness. Findings on non-human terrestrial species that do not display unihemispheric sleep are scarce and controversial. We are aware that our sample size with nineteen dogs limits the generalizability of our results, even if this sample is larger than what most research on humans (e.g.^9,46^) and non-human species (e.g.^52,56,57^) is based on.

A further aspect that makes direct comparisons between species difficult, is that data are collected using different methods (fMRI, MEG, EEG). Relatedly, slightly different EEG analysis are applied, including period-amplitude analysis (e.g.^47,48,58^) and spectral analysis (e.g.^46^). Furthermore, human studies also differ in which sleep EEG recordings are analysed. In some cases the second sleep recordings were used (e.g.^47,48^) to exclude the first-night effect^10,11^ but results are also reported based on first^58^ and even fourth and fifth sleep recordings^59^. Hereinafter it seems to be warranted to control for the number of sleep recordings.

Even so, in a recent study on humans (with the sample size of eleven right-handed subjects), a significant difference between the two hemispheres activity was observed on the first sleep session but not on the second. Furthermore, the degree of interhemispheric asymmetry was positively associated with the sleep-onset latency that is a sensitive marker of the first-night effect^9^. These results suggest that the first-night effect, which manifests in marked differences in sleep macrostructure between the first and second sleep occasions^10,11^, can be a consequence of one hemisphere being more vigilant than the other during sleep in an attempt to monitor the unfamiliar environment^9^. These results support the theory about the brain’s monitoring function in humans, which is consistent with the presumed function in aquatic mammals and birds^7^. If we assume that the protected environment shaped the characteristics of human sleep (including interhemispheric asymmetries), we might assume that similar changes occur in the sleep of the family dog that might have adapted to the protected human environment.

We found no clear association between hemispheric asymmetry and first night effect (FNE) in dogs in the current dataset. The lack of association might indicate that the leftward hemispheric asymmetry during the first sleep is not driven by the new test situation (sleeping in the laboratory with electrodes). It needs to be noted that dogs slept during the afternoon on non-consecutive days and they varied in their sleep habits (frequency of sleeping away from home in the presence of the owner), which seems to affect FNE-like adaptation processes in dogs^30^.

We can not exclude that the relatively long time intervals between the measurements interfered with our results, although in our previous study we did not find statistical evidence for the effect of the elapsed time between occasions affecting the FNE-like adaptation processes in dogs^30^. In humans FNE was explored not just on consecutive days (e.g.^10,11^) but also with longer time intervals. Lorenzo and Barbanoj^60^ collected data on twelve nights, with a minimum of one months between three periods, and one period consisted of 4 consecutive nights. They found that the FNE was only present in the first night of the first period (called the “very first night”).

Furthermore, it is possible that sleep habits had an effect on interhemispheric asymmetry but, due to the unbalanced distribution of dogs with experience sleeping not at home in the current sample, this effect could not be analysed. Sleep habits might have somewhat masked the association between the degree of asymmetry and FNE. One adaptation-like pattern that was observable in the current data relates to the finding of greater asymmetry in the first cycle of sleep recording 1, compared to the first cycle of sleep recording 2. However, considering absolute asymmetry, an opposite pattern was observable: in sleep recording 1, the second sleep cycle showed greater asymmetry in the alpha, sigma and beta frequency ranges, compared to the first sleep cycle. Similar results were detected in sleep recording 2. To our knowledge, in most human studies regarding hemispheric asymmetry, sleep cycles were not differentiated (except from^9^ where only the first sleep cycle was analysed).

With the present study, we provided evidence on interhemispheric differences in the natural sleep of the dog. To further explore the phenomenon of hemispheric asymmetry in dogs, experimental modulation (e.g. sleep pressure) could be applied to investigate sleep physiological effects such as sleep homeostasis, similarly to studies in humans^49,50^ and rats^54^.

To sum up, for the first time family dogs’ hemispheric asymmetry was investigated based on afternoon sleep recordings in an unfamiliar location. We provide solid evidence of hemispheric asymmetry during sleep in a domestic species, the dog, however, our findings may not generalize to sleep at nighttime and/or sleep after low activity. Despite the considerable individual differences regarding dogs’ sleep EEG spectrum asymmetry, we found a population level bias to the left during the first sleep recording. Differences between sleep recordings and consecutive sleep cycles are indicative of adaptation-like processes but do not closely resemble the first-night effect described in humans.

## Supplementary Information

Supplementary Information.
