# Supplementary material for: Interhemispheric asymmetry during NREM sleep in the dog
Source: Sci Rep. 2021 Sep 22;11:18817. doi: 10.1038/s41598-021-98178-3 (PMC8458274; doi:10.1038/s41598-021-98178-3)
Supplement: Supplementary file 1 — Supplementary Information. [file 41598_2021_98178_MOESM1_ESM.docx]

# SUPPLEMENTARY INFORMATION to:

#

#

#

#

#

#

Interhemispheric asymmetry during NREM sleep in the dog

Vivien Reicher^1,2^, Anna Kis^3^, Péter Simor^4,5^, Róbert Bódizs^5,6^, and Márta Gácsi^1,2^

^1^ Institute of Biology, Eötvös Loránd University, Department of Ethology, Budapest, Hungary

^2^ MTA-ELTE Comparative Ethology Research Group, Budapest, Hungary

^3^Institute of Cognitive Neuroscience and Psychology, Research Centre for Natural Sciences, Budapest, Hungary

^4^ Institute of Psychology, Eötvös Loránd University, Department of Ethology, Budapest, Hungary

^5^ Institute of Behavioural Sciences, Semmelweis University, Budapest, Hungary

^6^ Pázmány Péter Catholic University, Faculty of Humanities and Social Sciences, Budapest, Hungary


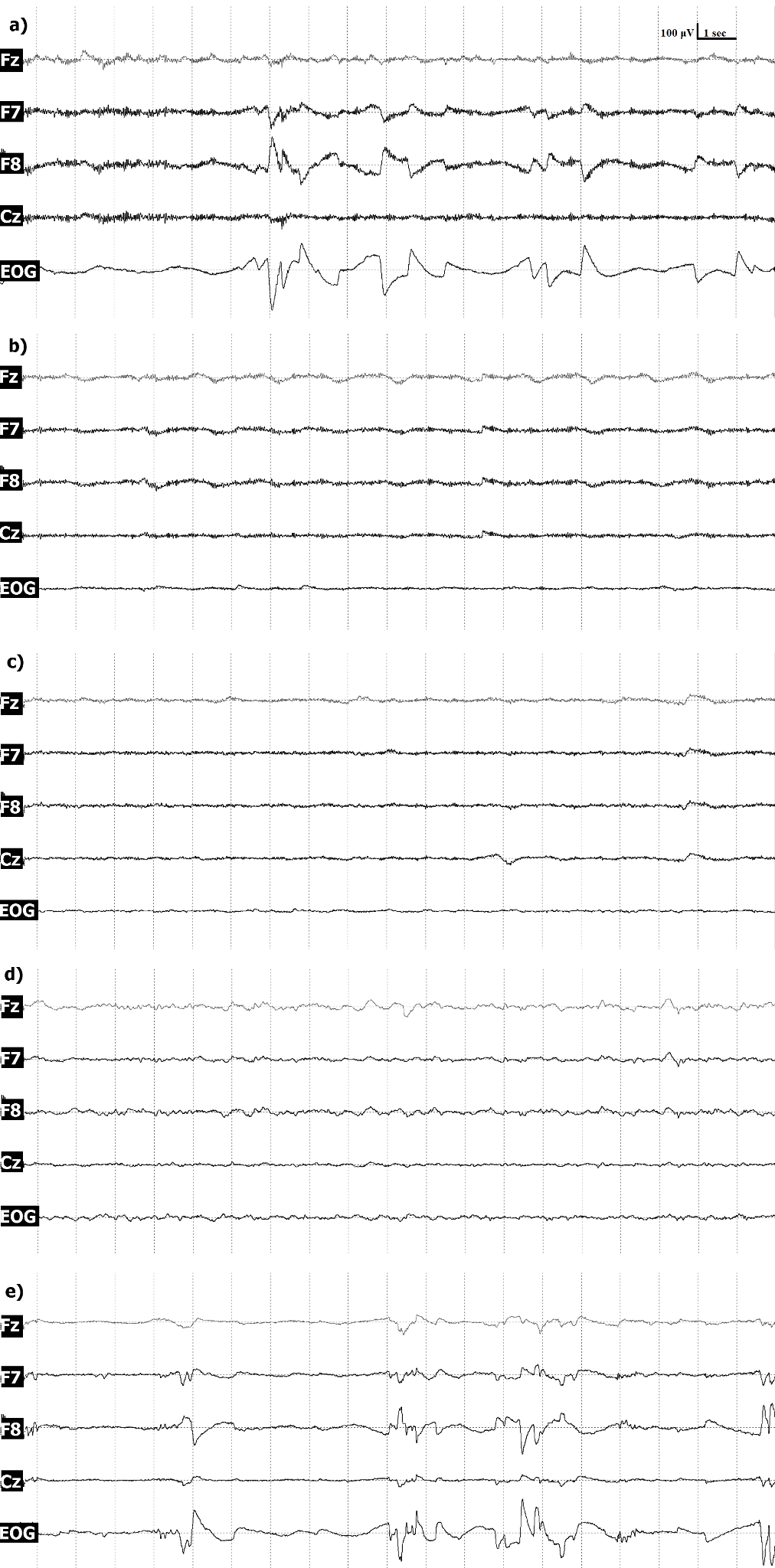


Fig. S1: EEG traces of sleep stages of a) wake, b) drowsiness with artefact, c) drowsiness without artefact, d) NREM, and e) REM.

Table S1:

*The mean +/ SD values of macrostructure variables. All sleep variables are given in minutes (min).*

| **Sleep recording** | **Sleep cycle** | **Drowsiness** | **NREM** | **REM** |
| --- | --- | --- | --- | --- |
| **1** | **1** | *9.45 ± 4.63* | *23.75 ± 10.19* | *3.08 ±2.94* |
| **1** | **2** | *8.06 ± 4.27* | *17.15 ± 13.22* | *5.20 ± 2.94* |
| **2** | **1** | *9.58 ± 6.03* | *21.37 ± 13.65* | *3.12 ± 2.97* |
| **2** | **2** | *7.02 ± 6.57* | *14.67 ± 13.58* | *5.44 ± 2.93* |

Table S2:

*Descriptive statistics on the comparison of sleep macrostructure data (Drowsiness, NREM and REM) in different sleep sessions and sleep cycles. We controlled for multiple comparisons with Benjamini-Hochberg correction (corr p).*

| ***Comparisons*** | | ***Drowsiness*** | | | ***NREM*** | | | ***REM*** | | |
| --- | --- | --- | --- | --- | --- | --- | --- | --- | --- | --- |
| *Sleep recording* | *Sleep cycle* | *t* | *p* | *corr p* | *t* | *p* | *corr p* | *t* | *p* | *corr p* |
| ***1*** | ***1 vs. 2*** | *0.589* | *0.564* | *0.845* | *1.874* | *0.078* | *0.313* | *-1.682* | *0.111* | *0.333* |
| ***2*** | ***1 vs. 2*** | *1.359* | *0.192* | *0.46* | *2.308* | *0.034* | *0.203* | *-2.399* | *0.028* | *0.203* |
| ***1 vs. 2*** | ***1*** | *-0.084* | *0.934* | *0.972* | *1.042* | *0.311* | *0.622* | *-0.035* | *0.972* | *0.972* |
| ***1 vs. 2*** | ***2*** | *0.468* | *0.646* | *0.861* | *0.914* | *0.373* | *0.64* | *-0.182* | *0.857* | *0.972* |


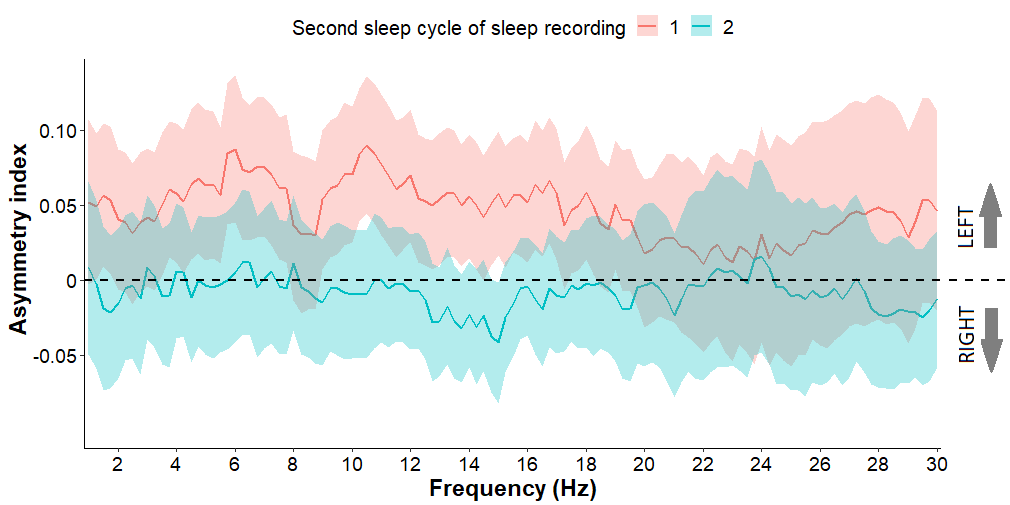


Fig. S2: Asymmetry index values (Mean +/- SE) in frequency range 1-30 Hz in the second sleep cycles of sleep recording 1 and 2. The second sleep cycle of sleep recording 1 and 2 did not deviate from the baseline level of zero asymmetry and we observed no difference between the second cycles of the two sleep recordings.


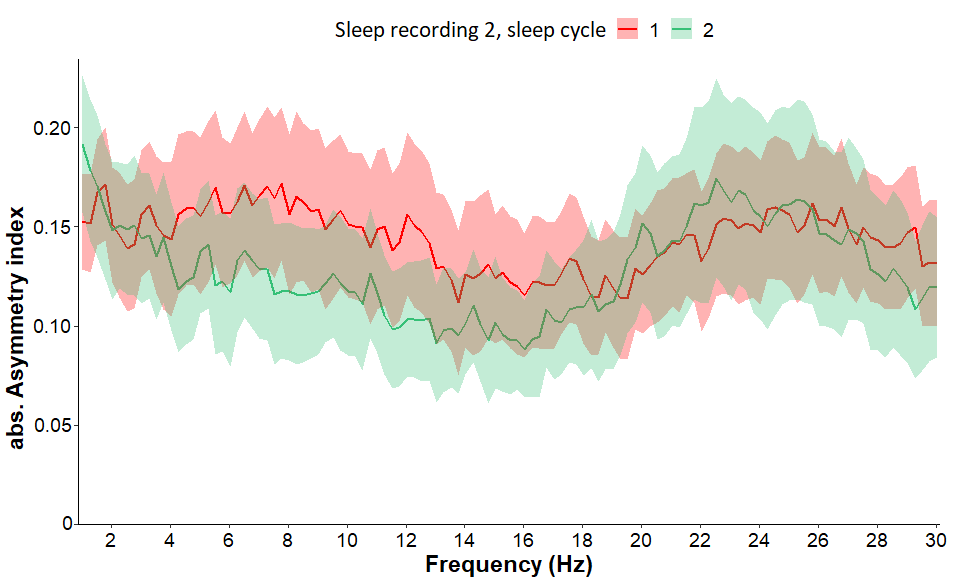


Fig. S3: Absolute asymmetry index values (Mean +/- SE) in frequency range 1-30 Hz in the first and second sleep cycles of sleep recording 2. We observed no difference between the first and second sleep cycles of sleep recording 2.


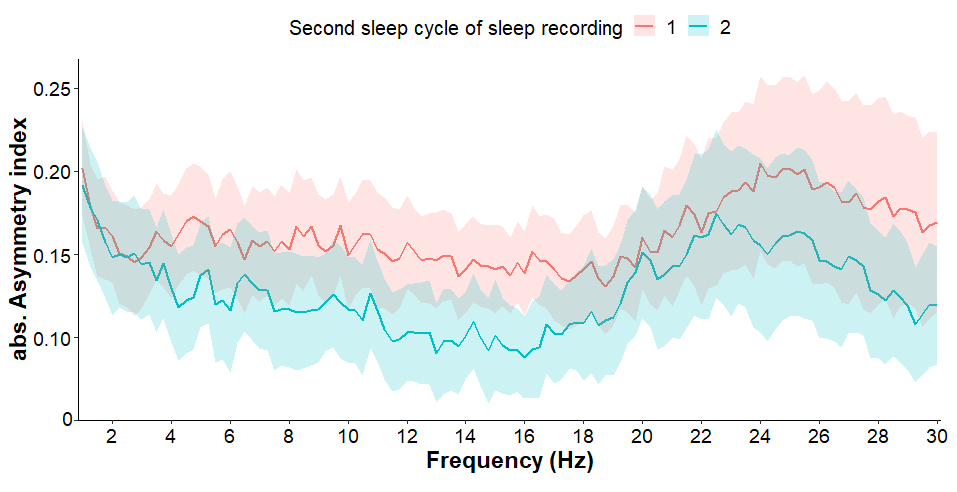


Fig. S4: Absolute asymmetry index values (Mean +/- SE) in frequency range 1-30 Hz in the first and second sleep cycles of sleep recording 2. We observed no difference between the second cycles of sleep recording 1 and 2.


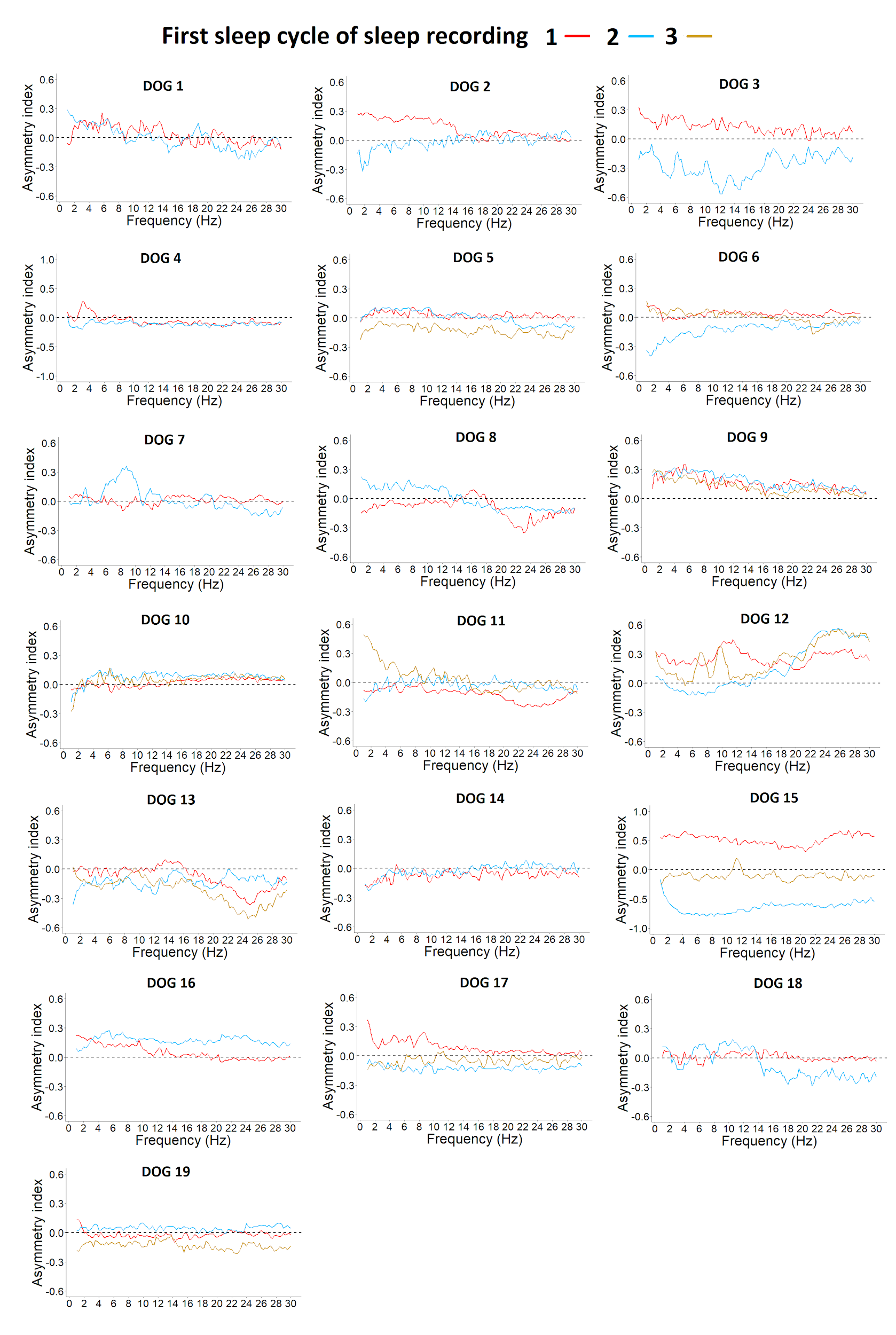


Fig. S5: Dogs’ individual asymmetry index values in the first sleep cycle of the two/three recordings.
